# Oxygen‐Doped MoS_2_ with Expanded Interlayer Spacing for Rapid and Stable Polysulfide Conversion

**DOI:** 10.1002/advs.202502834

**Published:** 2025-04-07

**Authors:** Wenqi Yan, Jinglin Xian, Shunan Zhang, Jiarui Zhang, Kaisi Liu, Jin‐Lin Yang, Feng Tao, Ruiping Liu, Qi Liu, Peihua Yang

**Affiliations:** ^1^ School of Materials Science and Engineering Anhui Polytechnic University Wuhu 241000 China; ^2^ The Institute of Technological Sciences MOE Key Laboratory of Hydrodynamic Transients Wuhan University Wuhan 430072 China; ^3^ Institute of Carbon Neutrality Shanghai Tech University Shanghai 201203 China; ^4^ Center for Computational Chemistry School of Chemistry and Chemical Engineering Wuhan Textile University Wuhan 430200 China; ^5^ School of Physical and Mathematical Sciences Nanyang Technological University Singapore 637371 Singapore; ^6^ Department of Materials Science and Engineering China University of Mining and Technology (Beijing) Beijing 100083 China

**Keywords:** Li–S batteries, MoS_2_, oxygen doping, expanded interlayer spacing, polysulfide conversion

## Abstract

Lithium–sulfur batteries face challenges such as the polysulfide shuttle effect and sluggish redox kinetics, leading to poor sulfur utilization and limited cyclic stability. Herein, an oxygen‐doped engineering approach is presented to achieve pillar‐free interlayer extension of MoS_2_ (E‐MoS_2_) for lithium polysulfide conversion. E‐MoS_2_ features expanded interlayer spacing (from 0.63 to 0.95 nm), improved conductivity, and an optimized Mo *d* band center, which collectively enhances polysulfide conversion efficiency. Consequently, cathodes with E‐MoS_2_ deliver a capacity of 638 mAh g^−1^ after 600 cycles at 2 C (0.046% decay/cycle) and an areal capacity of 12.0 mAh cm^−2^ under practical conditions (12 mg cm^−2^ S loading, E/S = 4 µL mg^−1^). This work highlights interlayer engineering as a key strategy for optimizing MoS_2_ catalysts in conversion‐type batteries.

## Graphical abstract

Oxygen‐doped, pillar‐free expanded MoS_2_ preserves the accessibility of catalytic edge sites while introducing additional lithium‐ion diffusion channels, facilitating stable and rapid polysulfide conversion. This work offers valuable insights for advancing Li–S batteries and holds broader implications for next‐generation energy storage technologies.

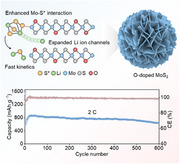

## 1 Introduction

Lithium–sulfur (Li–S) batteries have emerged as a promising candidate for next‐generation energy storage systems due to their remarkable theoretical energy density (≈2600 Wh kg^−1^), cost‐effectiveness from abundant sulfur reserves, and environmental sustainability.^[^ ^1^ ^]^ Practical Li–S configurations have already achieved energy densities exceeding 600 Wh kg^−1^, representing a 2–3 fold increase over conventional lithium‐ion batteries.^[^ ^2^, ^3^, ^4^, ^5^ ^]^ This superior energy storage capability positions Li–S technology as a transformative solution for applications ranging from high‐endurance drones to long‐range electric vehicles and grid‐scale energy storage.^[^ ^6^, ^7^, ^8^, ^9^ ^]^ However, challenges such as the insulating nature of sulfur cathodes, the detrimental polysulfide shuttling effect, and sluggish redox kinetics remain, leading to rapid capacity fading and low Coulombic efficiency (CE). While nanostructured carbon hosts (e.g., graphene aerogels, microporous carbons) have been explored to suppress shuttling, their nonpolar surfaces exhibit weak interactions with polar lithium polysulfides (LiPSs), resulting in leakage and severe capacity decay under lean‐electrolyte conditions.^[^ ^10^, ^11^ ^]^ Recent efforts have focused on chemisorption‐catalysis synergy, leveraging electrocatalysts to anchor LiPSs and accelerate their conversion, thereby suppressing shuttling and extending cycle life.^[^ ^12^, ^13^, ^14^ ^]^

2D transition metal dichalcogenides (TMDs), exemplified by MoS_2_, have gained prominence in electrocatalysis due to their unique layered structure and exceptional catalytic properties.^[^ ^15^, ^16^ ^]^ Previous investigations have shown that TMDs exhibit facet‐dependent LiPSs adsorption, with stronger metal‐S bonds at the edge compared to the weaker chalcogen‐Li bonds on the basal plane.^[^ ^17^, ^18^, ^19^, ^20^, ^21^ ^]^ However, MoS_2_ suffers from low conductivity and the scarcity of active edge sites, resulting in suboptimal catalytic performance. To address this, strategies such as nanostructuring and chemical doping have been developed to enhance edge site exposure and intrinsic activity. Additionally, expanding the (002) interlayer spacing through pillar‐insertion techniques has been employed to activate the basal planes and improve electrocatalytic performance. For instance, the integration of carbon layers and Fe atoms into MoS_2_ has formed conductive S─Fe─C networks, enhancing electron delocalization and promoting sulfur conversion.^[^ ^22^ ^]^ While other pillar systems, including organic molecules (e.g., CTAB),^[^ ^23^ ^]^ ionic species (e.g., SCN^–^),^[^ ^24^ ^]^ and graphene,^[^ ^25^ ^]^ have also shown promise in expanding the interlayer spacing, they inevitably hinder lithium ion diffusion. Therefore, pillar‐free interlayer expansion, combined with modulation of active sites and conductivity, could offer a viable approach toward stable and efficient sulfur conversion.^[^ ^26^ ^]^

**Figure 1:**
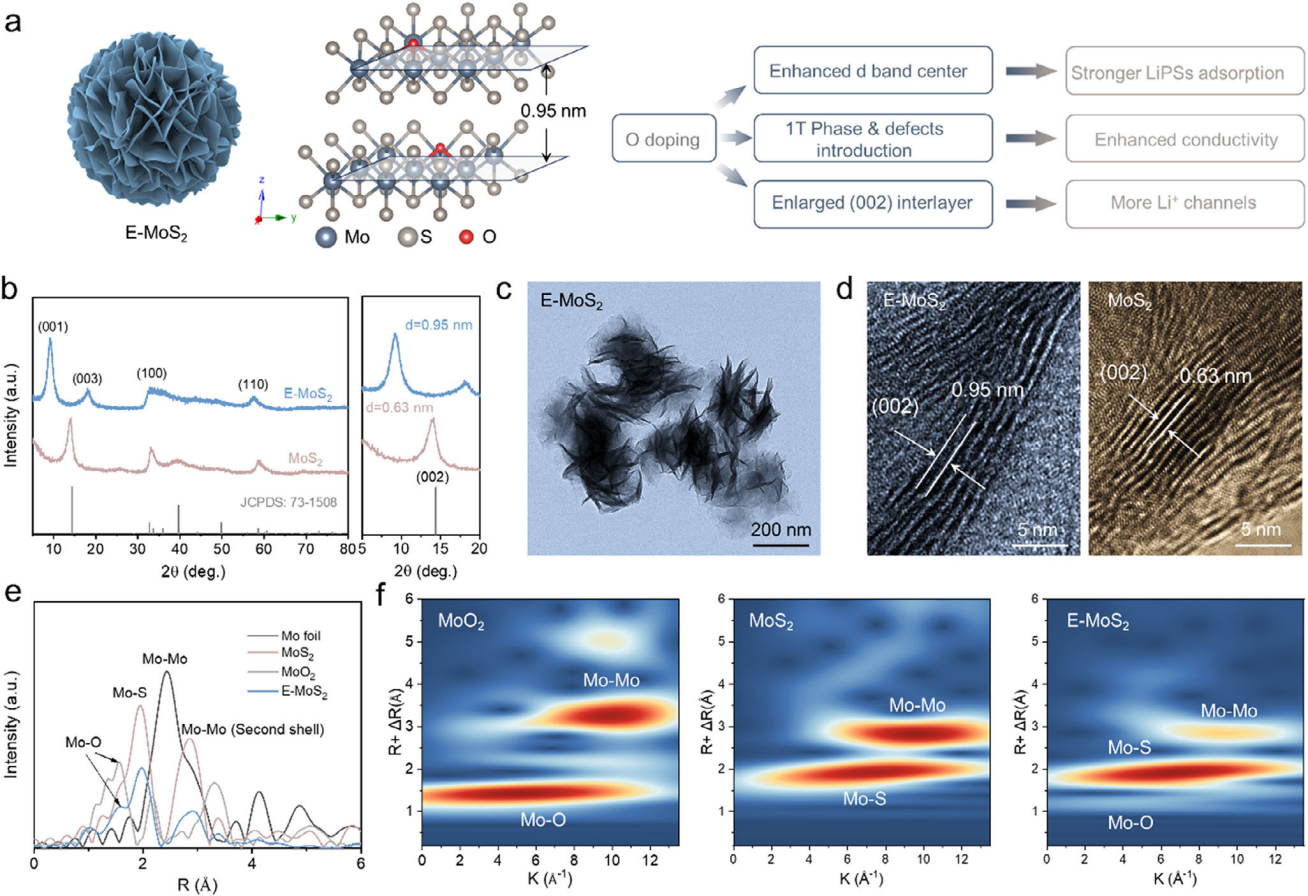
Interlayer engineering and structural characterization of E‐MoS_2_. a) Schematic of the E‐MoS_2_ crystal structure and the influence of O doping. b) XRD patterns, c) TEM, and d) HRTEM images. e) FT‐EXAFS spectra at the Mo K‐edge for the E‐MoS_2_, MoO_2_, MoS_2_, and Mo foil. f) WT‐EXAFS spectra of the MoO_2_, MoS_2_ and E‐MoS_2_.

Herein, we developed an oxygen‐doped MoS_2_ (E‐MoS_2_) catalyst through molecular engineering to enhance LiPSs conversion kinetics. The introduction of defects within the MoS_2_ lattice facilitates the basal plane activation. Oxygen doping induces interlayer Coulomb repulsion, expanding the (002) interlayer spacing from 0.63 to 0.95 nm, which preserves catalytic edge site accessibility while creating additional Li^+^ diffusion channels. This doping also modulates the Mo 3d orbital electronic structure, promoting the formation of a rich 1T‐phase and enhancing bulk conductivity. Furthermore, the strengthened *d*‐*p* hybridization between Mo and S optimizes LiPSs adsorption and reduces charge transfer barriers, improving the overall electrocatalytic performance of E‐MoS_2_ (**Figure** **1**a). Given these merits, Li─S batteries incorporating E‐MoS_2_ demonstrate enhanced specific capacity and cycling stability, even under high sulfur loading and lean electrolyte conditions. This work provides a defect‐heteroatom synergy strategy for activating TMDs, advancing catalytic material design for high‐energy‐density Li─S systems.

## 2 Results and Discussion

The pre‐intercalated MoS_2_ was achieved via a one‐step hydrothermal method, with a relatively low reaction temperature expanding the interlayer spacing from 0.63 nm (MoS_2_) to 0.95 nm (E‐MoS_2_) (Figure 1a). This expansion is attributed to the incomplete reaction between the molybdate and thiourea precursors.^[^ ^18^ ^]^ X‐ray diffraction (XRD) patterns show that the pristine MoS_2_ corresponds to the hexagonal 2H‐phase, while E‐MoS_2_ exhibits characteristic (001) and (003) peaks at 2θ = 9.14° and 18.05°, respectively, with a two−fold relationship and the disappearance of the (002) peak (Figure 1b), consistent with 1T‐phase MoS_2_.^[^ ^23^, ^27^ ^]^ The expanded interlayer spacing of E‐MoS_2_, results from shortened Mo−O bonds and sulfur vacancies, which weaken the van der Waals interactions between adjacent layers.^[^ ^28^, ^29^ ^]^

The synthesized E‐MoS_2_ consists of ultrathin wrinkled nanosheets with a uniform lateral size of 200–300 nm, as verified by scanning electron microscopy (SEM) and transmission electron microscopy (TEM) (Figure 1c; Figure S1, Supporting Information). The observed ripples and corrugations in the nanosheets highlight their ultrathin nature, which promotes the exposure of catalytically active sites and facilitates efficient LiPSs conversion. Scanning TEM (STEM) and elemental mapping of E‐MoS_2_ confirm the presence of oxygen (Figure S2, Supporting Information). Additionally, high‐resolution TEM (HRTEM) images of the curled nanosheet edges reveal a notably increased layer spacing of 0.95 nm for E‐MoS_2_ compared to 0.63 nm for MoS_2_, consistent with the XRD results (Figure 1d). A comparison of various expansion strategies highlights that oxygen doping in E‐MoS_2_ achieves a substantial interlayer spacing of 0.95 nm (Table S1, Supporting Information), comparable to conventional expansion methods but without obstructing ion transport channels. Furthermore, nitrogen adsorption‐desorption isotherms demonstrate that the specific surface area of E‐MoS_2_, increases from 24.0 to 45.6 m^2^ g^−1^, while the pore size remains similar to that of pristine MoS_2_, correlating with the observed interlayer spacing expansion (Figure S3, Supporting Information).

The electronic state and coordination environment of Mo atoms in E‐MoS_2_ were further analyzed using Mo K‐edge X‐ray absorption near‐edge structure (XANES) and extended X‐ray absorption fine structure (EXAFS). The Mo K‐edge XANES spectra show that the absorption edge position of E‐MoS_2_ lies between those of MoS_2_ and MoO_2_, indicating a reduction in the oxidation state of Mo atoms and an increase in electron density due to charge redistribution from oxygen incorporation (Figure S4, Supporting Information). The corresponding Fourier‐transform EXAFS (FT‐EXAFS) spectra exhibit a distinct peak at ≈1.6 Å (Figure 1e), confirming Mo‐O coordination and partial substitution of Mo─S with Mo─O bonds. The higher electronegativity of oxygen atoms induces polarization of Mo's *d*‐electron density, leading to enhanced positive charge on Mo centers, consistent with the electronic modulation observed in XANES analysis.^[^ ^30^ ^]^ The K‐edge wavelet transform EXAFS (WT‐EXAFS) spectra of E‐MoS_2_ show three distinct maxima at 6.0, 6.7, and 9.4 Å^−1^, corresponding to Mo‐O, Mo‐S, and Mo‐Mo coordination, respectively (Figure 1f). Attenuated Mo‐Mo scattering versus controls align with HR‐TEM confirmed interlayer expansion.^[^ ^31^, ^32^ ^]^ The coexistence of Mo‐O and Mo‐S coordination creates dual electron transfer pathways: oxygen polarization enhances charge redistribution, while the retained Mo‐S framework preserves basal conductivity. This electronic modulation optimizes charge transfer at active sites, potentially enhancing LiPSs redox kinetics.

**Figure 2:**
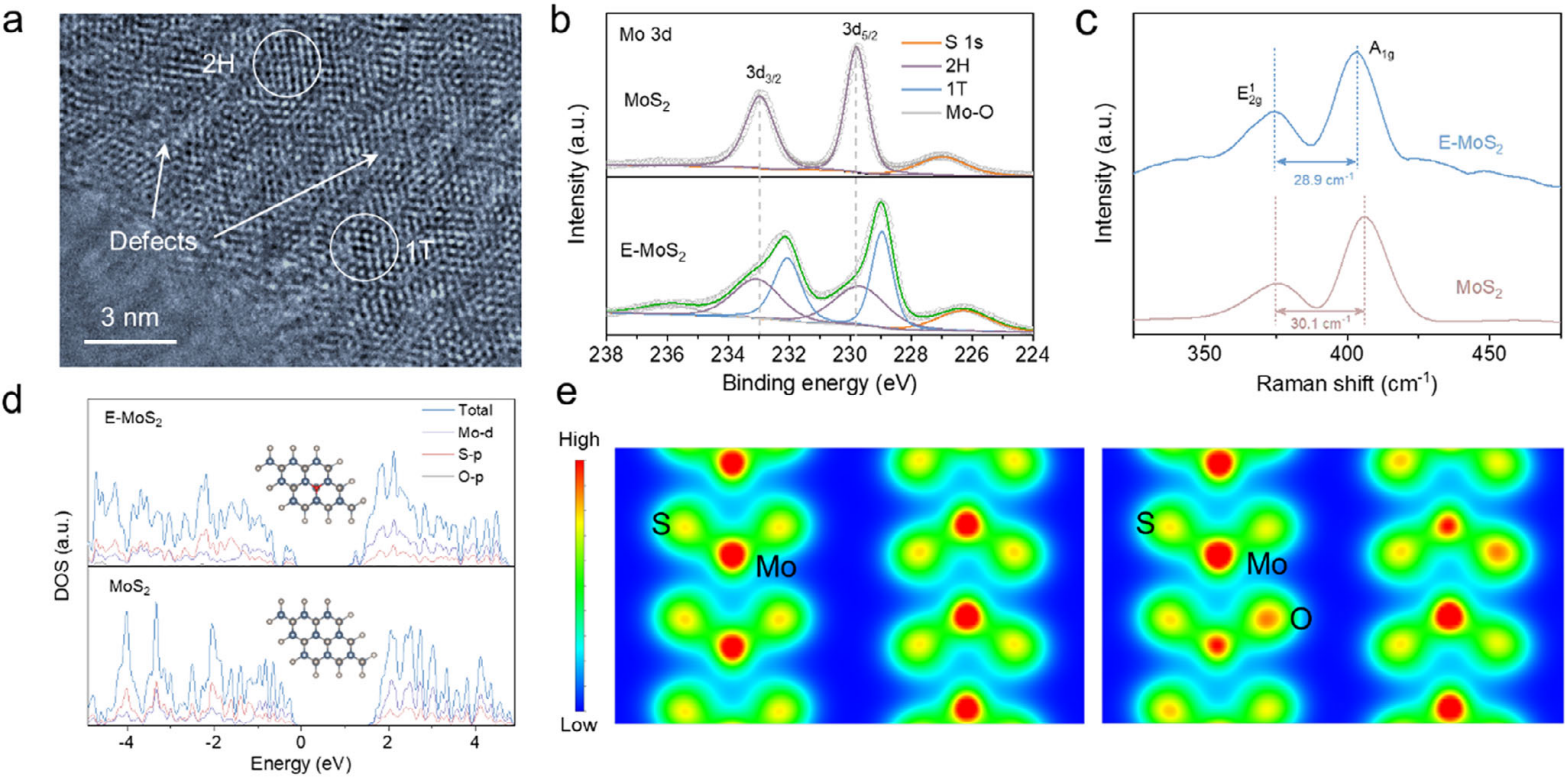
Microstructure and electronic structure characterization of E‐MoS_2_. a) HAADF‐TEM. b) Mo 3d XPS spectra, and c) Raman spectra. d) Calculated DOS of the E‐MoS_2_ slab (top panel) and MoS_2_ slab (bottom panel). e) Charge density distribution.

High‐angle annular dark field TEM (HAADF‐TEM) images reveal abundant 1T‐phase trigonal lattices and sparse 2H‐phase honeycomb lattices in E‐MoS_2_ (**Figure** **2**a). The dominance of the 1T‐phase, combined with defects and oxygen incorporation, contributes to enhanced conductivity. The electron paramagnetic resonance (EPR) spectra show a pronounced Mo‐S dangling bond signal at g = 2.003 for E‐MoS_2_ (Figure S5, Supporting Information), significantly stronger than that of pristine MoS_2_, indicating a higher concentration of sulfur vacancies in the E‐MoS_2_ structure. X‐ray photoelectron spectroscopy (XPS) analysis further supports this, with characteristic peaks of E‐MoS_2_ shifting toward lower binding energies compared to pristine MoS_2_ (Figure 2b; Figure S6, Supporting Information), confirming the presence of S‐vacancies.^[^ ^33^, ^34^ ^]^ The Mo 3d spectrum consists of two doublets assigned to Mo^4+^ 3d_5/2_ and Mo^4+^ 3d_3/2_, with spectral deconvolution revealing a transition from predominantly 2H‐phase in pristine MoS_2_ to 1T‐phase dominance in E‐MoS_2_. Raman spectra offer complementary structural insights (Figure 2c). The $E_{2g}^{1}$ mode of E‐MoS_2_ exhibits a redshift and peak broadening relative to pristine MoS_2_, suggesting softening of the basal plane Mo‐S bonds.^[^ ^28^ ^]^ The reduced $E_{2g}^{1}$‐A_1_ _g_ peak separation reflects weakened interlayer interactions, consistent with the expanded interlayer spacing observed in XRD and TEM.

Oxygen incorporation in MoS_2_ is an effective method for modulating the electronic structure and improving intrinsic conductivity.^[^ ^18^, ^35^ ^]^ First‐principles calculations were performed to analyze the effect of oxygen doping on the electronic structure of MoS_2_. The calculated density of states (DOS) demonstrates a reduced bandgap of 1.07 eV for E‐MoS_2_ compared to 1.54 eV for pristine MoS_2_ (Figure 2d), suggesting increased charge carrier density and enhanced conductivity of the E‐MoS_2_ nanosheets. This bandgap narrowing arises from strengthened Mo *d*‐orbital and S *p*‐orbital hybridization induced by oxygen doping. Charge density distribution analysis further visualizes oxygen doping's effects, with significant charge accumulation at oxygen dopants in E‐MoS_2_, confirming the modification of the electronic structure (Figure 2e). These results suggest that oxygen doping improves intrinsic conductivity and benefits ion transport for LiPSs conversion electrocatalysis.

**Figure 3:**
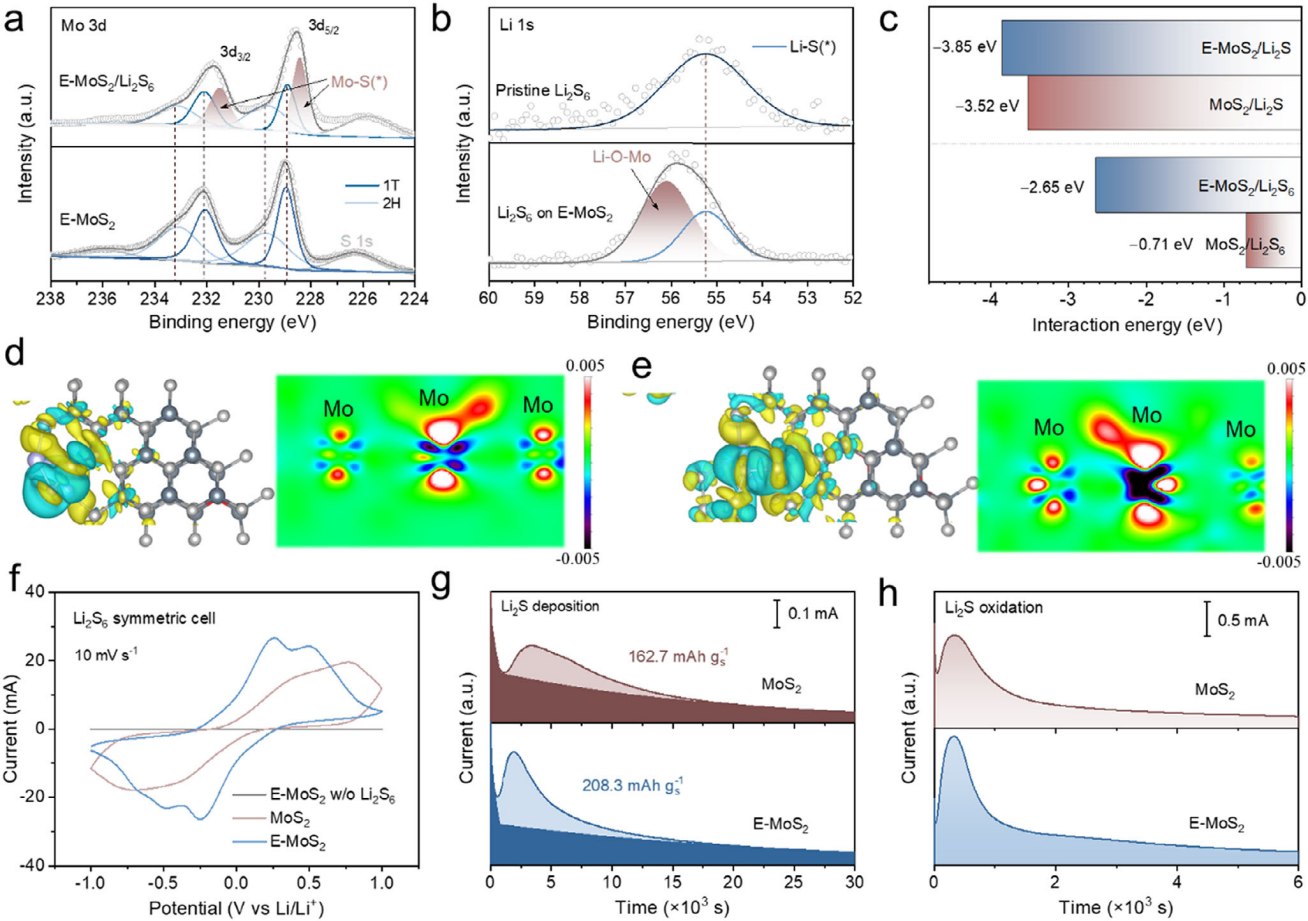
Sulfur species interaction and conversion with E‐MoS_2_. a) Mo 3d and b) Li 1s XPS spectra before and after Li_2_S_6_ adsorption. c) Interaction energy between sulfur species (Li_2_S and Li_2_S_6_) and catalysts. Electron‐density difference of d) Li_2_S and e) Li_2_S_6_ on the surface of E‐MoS_2_. f) CV curves of Li_2_S_6_ symmetric cells at a scan rate of 10 mV s^−1^. Potentiostatic nucleation profiles of g) Li_2_S deposition and h) Li_2_S dissolution on MoS_2_ and E‐MoS_2_ electrodes.

Before the electrochemical performance characterizations, ultraviolet–visible (UV–vis) spectroscopy was employed to evaluate the Li_2_S_6_ adsorption capabilities of samples (Figure S7, Supporting Information). A characteristic peak ≈ 260 nm, associated with S_6_^2−^/S_8_^2−^ species,^[^ ^8^ ^]^ demonstrated remarkable attenuation after E‐MoS_2_ treatment compared to the pristine Li_2_S_6_ solution. Notably, the solution with E‐MoS_2_ exhibited substantially reduced absorbance relative to both graphene (G) and MoS_2_, confirming the superior LiPSs adsorption capacity. To further investigate the chemical interaction mechanism of Li_2_S_6_ on E‐MoS_2_, XPS results were collected after the adsorption test. The Mo 3d spectra show a characteristic doublet at 228.6 eV (Mo 3d_5/2_) and 231.7 eV (Mo 3d_3/2_) in E‐MoS_2_ after Li_2_S_6_ adsorption, which shifts negatively by ≈0.4 eV relative to E‐MoS_2_ (**Figure** **3**a). This shift suggests electron transfer from Mo to sulfur species, forming Mo‐S bonds through Lewis acid‐base interactions.^[^ ^36^ ^]^ Additionally, a Li‐O‐Mo interaction was observed in the Li 1s spectrum (Figure 3b). These findings suggest that E‐MoS_2_ facilitates strong chemical adsorption of LiPSs.

Density functional theory (DFT) calculations were performed to assess the interaction energies between samples and LiPSs (Figure 3c; Figure S8, Supporting Information), focusing on representative species (Li_2_S and Li_2_S_6_). Both E‐MoS_2_ and MoS_2_ showed negative adsorption energies toward Li_2_S/Li_2_S_6_, indicating thermodynamically favorable adsorption. Notably, E‐MoS_2_ exhibited significantly stronger binding, as reflected by its more negative adsorption energies (−2.65 eV for Li_2_S_6_ and −3.85 eV for Li_2_S) compared to MoS_2_ (−0.71 and −3.52 eV, respectively). To further investigate the atomic‐scale interactions, the charge density difference between the catalysts and sulfur species was simulated. Comparative analysis revealed enhanced charge density redistribution in the E‐MoS_2_/Li_2_S and E‐MoS_2_/Li_2_S_6_ systems compared to their MoS_2_ counterparts (Figure 3d,e; Figure S9, Supporting Information). This pronounced charge accumulation suggests stronger electronic coupling at the E‐MoS_2_ interface and confirms its superior chemisorption capability.

To probe the redox kinetics of LiPSs, symmetric cells with Li_2_S_6_‐containing electrolytes were assembled. Electrodes were fabricated by coating carbon paper with E‐MoS_2_ and MoS_2_ catalysts. Cyclic voltammetry (CV) at 10 mV s^−1^ revealed reduced peak polarization for the E‐MoS_2_ electrode (Figure 3f), implying enhanced electrocatalytic activity toward LiPSs conversion. Li_2_S nucleation experiments further quantified the reaction kinetics (Figure 3g). According to Faraday's law,^[^ ^37^, ^38^ ^]^ the Li_2_S deposition capacity reached 208.3 mAh g^−1^ on E‐MoS_2_, compared to 162.7 mAh g^−1^ on MoS_2_, reflecting the higher density of active sites in E‐MoS_2_. While MoS_2_ exhibited nonuniform Li_2_S particle distribution due to limited nucleation sites, E‐MoS_2_ facilitated the formation of a thick, rough Li_2_S layer, suggesting altered growth kinetics (Figure S10, Supporting Information). The dissolution kinetics of Li_2_S were also evaluated by monitoring oxidation currents (Figure 3h). The E‐MoS_2_ electrode exhibited higher oxidation current density than MoS_2_, suggesting faster Li_2_S dissolution kinetics in the absence of catalytic confinement. These results show that the E‐MoS_2_ significantly enhances the redox kinetics, facilitating both the reduction of LiPSs and subsequent oxidation of Li_2_S.

**Figure 4:**
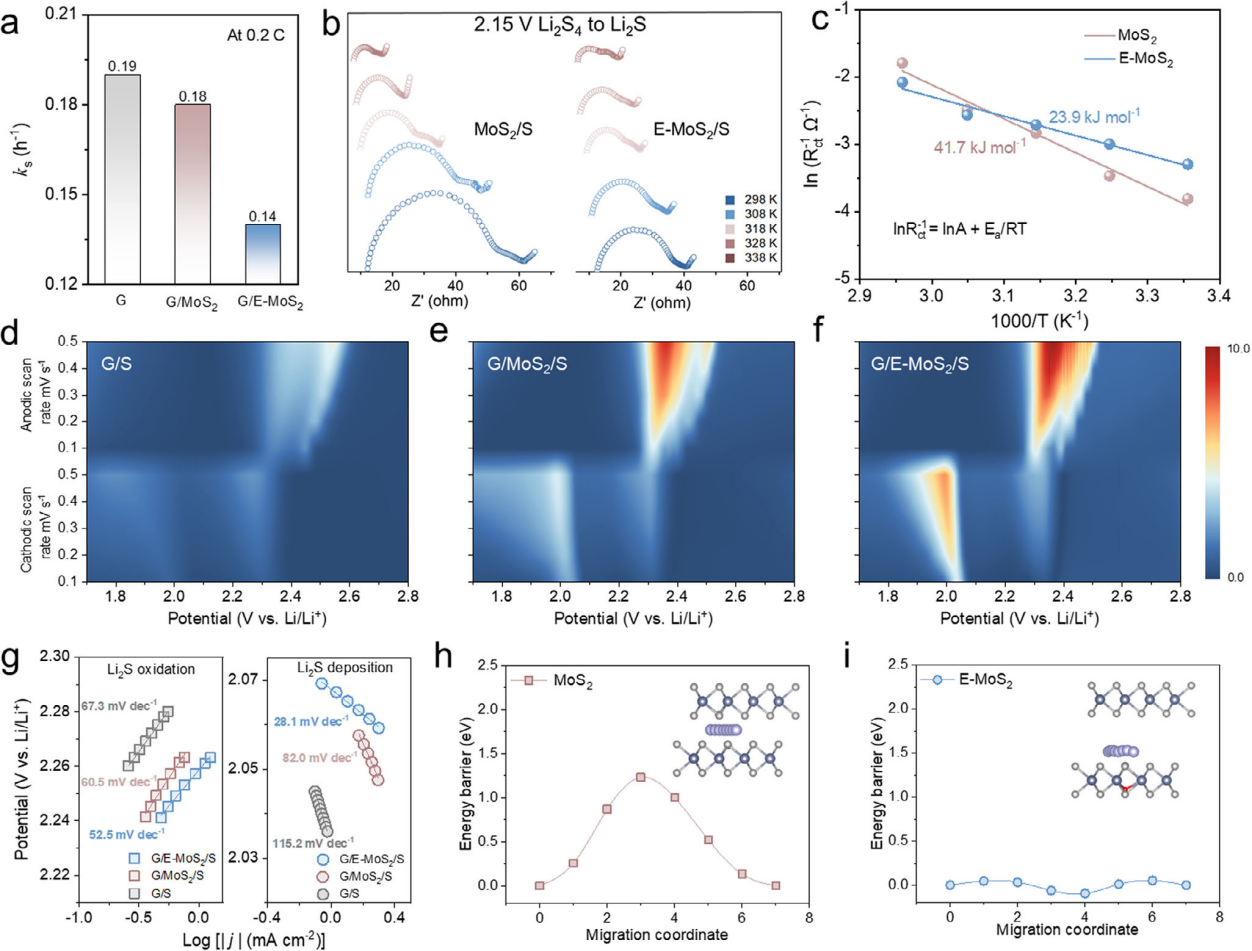
Electrocatalysis behavior demonstration. a) Shuttle constants for various batteries, presented as mean values. b) EIS measurements at 2.15 V, and c) the derived Arrhenius plots. Contour plots of CV patterns for d) G, e) MoS_2,_ and f) E‐MoS_2_. g) Tafel plots of Li_2_S oxidation and Li_2_S deposition. Energy profiles for the diffusion process of Li^+^ on h) MoS_2_ and i) E‐MoS_2_. Insets present a side‐view of the corresponding diffusion pathways.

The shuttle constant (*k*_s_) is a parameter used to evaluate the stability of charged electrodes, derived from charge efficiency variations at the high‐voltage plateau (**Figure** **4**a). For the G/E‐MoS_2_ catalyst, *k*_s_ was calculated to be 0.14, suggesting efficient oxidation of discharge products.^[^ ^39^ ^]^ In comparison, significantly higher values were observed for G/MoS_2_ (0.18) and G electrodes (0.19), implying greater overpotential requirements for LiPSs oxidation. To assess lithiation kinetics, the activation energy (*E*_a_) for the Li_2_S_4_ to Li_2_S conversion step was analyzed by recording charge‐transfer resistances at 2.15 V (Figure 4b). The Arrhenius plots revealed a linear relationship between the inverse of absolute temperature and the logarithm of the reciprocal of the charge‐transfer resistance. The *E*_a_ value for E‐MoS_2_ (23.9 kJ mol^−1^) was substantially lower than that of MoS_2_ (41.7 kJ mol^−1^) (Figure 4c), indicating enhanced catalytic activity in electrochemical tests. This reduction in *E*_a_ promotes solid Li_2_S nucleation, accelerates sulfur redox kinetics, and improves polysulfide conversion efficiency. Consequently, E‐MoS_2_ mitigates the polysulfide shuttle effect, enhancing sulfur utilization, extending cycle life, and improving the overall electrochemical performance of Li–S batteries.

CV analysis of the G/S composite cathode with various catalysts was conducted to investigate the sulfur reduction kinetics under scanning rates from 0.1–0.5 mV s^−1^ over 1.7−2.8 V (vs Li/Li^+^) (Figures S11,S12, and Table S2, Supporting Information). The battery with G/E‐MoS_2_ exhibited sharper redox peaks and reduced voltage polarization compared to MoS_2_/G and G at 0.1 mV s^−1^, suggesting enhanced sulfur redox kinetics and high reversibility. Contour plots of the CV patterns for G, MoS_2,_ and E‐MoS_2_ were presented in Figure 4d–f. The cathode with E‐MoS_2_ showed superior current response and narrower voltage polarization than G and MoS_2_ across all scan rates.

Linear relationships between peak currents and the square root of scan rates confirmed that both Li_2_S deposition and oxidation processes are diffusion‐limited (Figure S12, Supporting Information). Analysis using the Randles‐Sevcik equation indicated that the Li^+^ diffusion coefficients, as reflected by the slope values of fitted curves, are higher for E‐MoS_2_ compared to MoS_2_,^[^ ^40^ ^]^ which aligns with its superior ion transport properties. Additionally, Tafel plots of Li_2_S deposition/oxidation processes further evaluated electrocatalytic activities during LiPSs conversion (Figure 4g). Cells with E‐MoS_2_ exhibited lower Tafel slope values (52.5 and 28.1 mV dec^−1^) compared to MoS_2_ (60.5 and 82.0 mV dec^−1^) and G (67.3 and 115.2 mV dec^−1^), indicating improved bi‐directional electrocatalysis activity. This enhanced performance is attributed to E‐MoS_2_’s strong LiPSs chemisorption and rapid reaction kinetics.

Given the critical role of Li^+^ in LiPSs conversions, Li^+^ diffusion kinetics were calculated (Figure S13, Supporting Information). The calculated diffusion barrier for Li^+^ migration in E‐MoS_2_ (0.05 eV) was substantially lower than that in MoS_2_ (1.23 eV) (Figure 4h,i), suggesting facilitated Li^+^ transport through the E‐MoS_2_ interlayer structure. This reduced energy barrier correlates with accelerated LiPSs conversion kinetics, as enhanced Li^+^ mobility promotes charge transfer during redox reactions.

**Figure 5:**
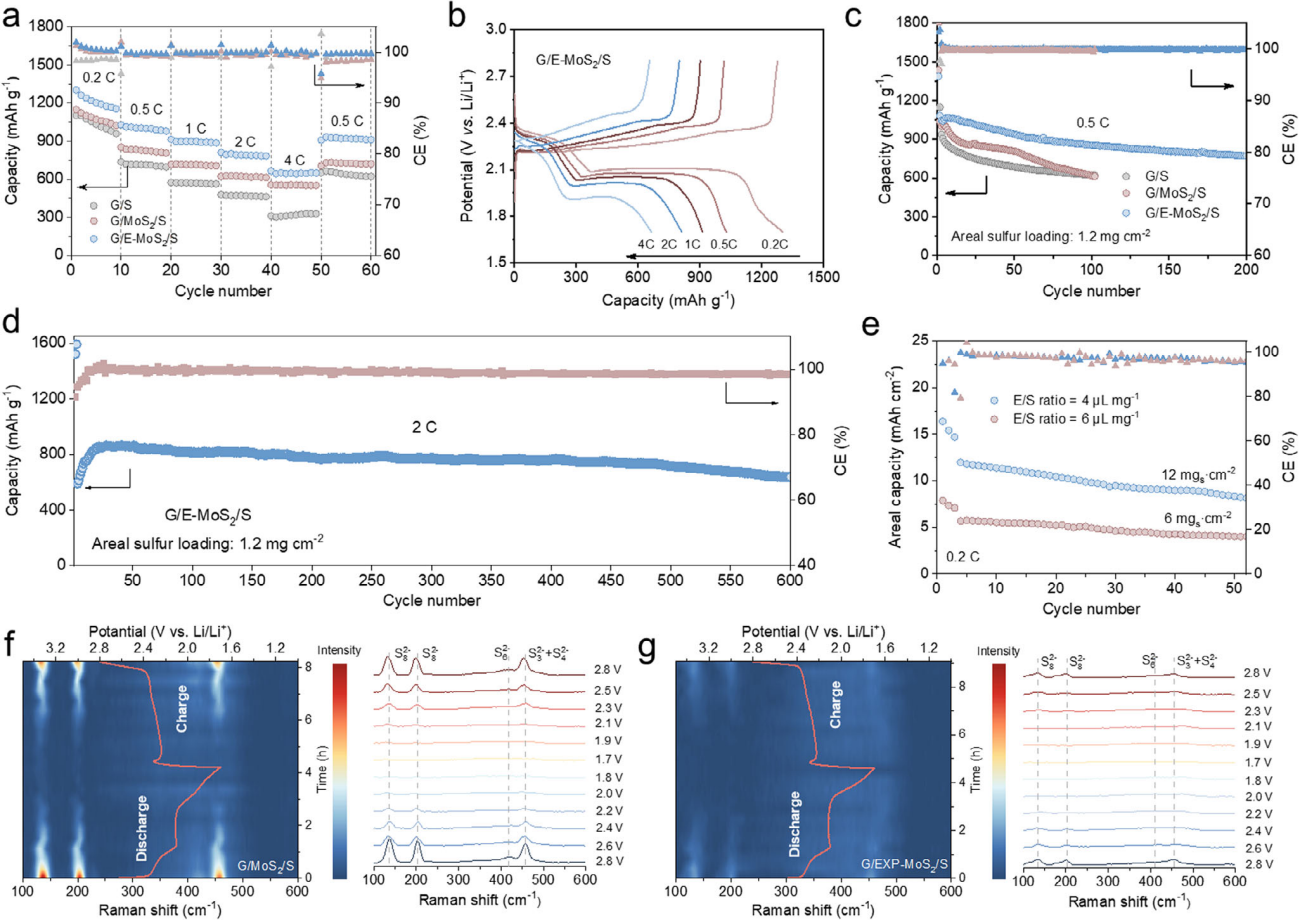
Electrochemical behavior of E‐MoS_2_ catalyst. a) Rate capability. b) Charge‐discharge curves. c) Long‐term cyclability of batteries with different catalysts in sulfur cathode at 0.5 C (1 C = 1675 mAh g^−1^). d) Long‐term cycling performance at 2 C. e) Cycling performance of batteries with high sulfur loading. In situ Raman contour plots and spectra of batteries with f) MoS_2_ and g) E‐MoS_2_.

The electrochemical performance of Li–S batteries was evaluated at current densities from 0.2 to 4 C within the voltage window of 1.7 to 2.8 V at 25 °C (**Figure** **5**a). The E‐MoS_2_‐based battery exhibited a lower voltage polarization (150 mV at 0.2 C) compared to MoS_2_ (180 mV, Figure S14, Supporting Information). The insets further illustrate the reduced activation and nucleation energy barriers for Li_2_S in E‐MoS_2_ system, which enhance reaction kinetics, sulfur utilization, and capacity performance. While capacities of MoS_2_‐based batteries decreased significantly with increasing current density, the E‐MoS_2_ system maintained high capacities of 1302, 1029, 913, 812, and 665 mAh g^−1^ at 0.2, 0.5, 1, 2, and 4 C, respectively, outperforming the control cells. Voltage profiles further corroborate these trends (Figure 5b). E‐MoS_2_ batteries retained well‐defined oxidation/reduction plateaus across all rates, indicative of stable sulfur redox processes. In contrast, cells with MoS_2_ exhibited incomplete redox plateaus at 1 C or higher (Figure S15, Supporting Information).

Long‐term cycling tests at 0.5 C revealed distinct performance differences (Figure 5c). The battery with E‐MoS_2_ catalyst delivered an initial capacity of 1086 mAh g^−1^, retaining 773 mAh g^−1^ after 200 cycles with a capacity decay rate of 0.14% per cycle. In contrast, batteries with MoS_2_ and G exhibited lower capacities with rapid degradation within 100 cycles. At a higher discharge rate of 2 C, the E‐MoS_2_ cell exhibited exceptional cycling stability, retaining 638 mAh g^−1^ after 600 cycles with a Coulombic efficiency of 98.6% and a capacity decay rate of 0.046% per cycle (Figure 5d). Further evaluations under high sulfur loadings and lean electrolyte conditions demonstrated the practical applicability of E‐MoS_2_ (Figure 5e). After three activation cycles at 0.1 C, the battery achieved an initial areal capacity of 5.7 mAh cm^−2^ at 0.2 C with a sulfur loading of 6 mg cm^−2^ and an E/S ratio of 6 µL mg^−1^. Under more stringent conditions (12 mg cm^−2^ sulfur loadings and an E/S ratio of 4 µL mg^−1^), the battery delivered an initial capacity of 12.0 mAh cm^−2^, retaining 8.2 mAh cm^−2^ after 50 cycles (Table S3, Supporting Information). This performance surpasses that of commercial Li‐ion batteries (typically ≈4 mAh cm^−2^), highlighting the practical viability of E‐MoS_2_ for high‐energy‐density applications.

To probe the catalytic conversion of LiPSs by E‐MoS_2_, in situ Raman spectroscopy was conducted to monitor the highly soluble LiPSs in the electrolyte during battery operation. Analysis of the diffusion of LiPSs toward the lithium anode showed that cathodes with MoS_2_ exhibited intense S_8_^2−^ peaks at 135.5 and 204.1 cm^−1^ at initial discharge, followed by persistent S_4_^2−^+ S_3_^2−^ signals during cycling (Figure 5f,g). The detection of these species suggests that LiPSs shuttling contributes to capacity loss. In contrast, E‐MoS_2_ cathodes displayed only transient S_4_^2−^+ S_3_^2−^ signals during both the discharge and charge processes, indicating effective suppression of LiPSs shuttling.

## 3 Conclusion

In summary, we developed an oxygen‐doped MoS_2_ catalyst with pillar‐free interlayer extension (from 0.63 to 0.95 nm) for Li–S batteries. Structural and theoretical analyses reveal that the elevated Mo *d*‐band center in E‐MoS_2_ strengthens LiPSs chemisorption through optimized orbital interactions. The 1T‐phase‐dominated structure, enriched with defects, boosts electrical conductivity while the expanded (002) basal planes provide abundant active sites and Li^+^ diffusion channels, facilitating efficient LiPSs conversion. Batteries incorporating E‐MoS_2_ exhibit exceptional cycling stability, retaining 638 mAh g^−1^ after 600 cycles at 2 C with a 0.046% capacity decay per cycle. Under high sulfur loading (12 mg cm^−2^) and lean electrolyte (E/S = 4 µL mg^−1^), the batteries deliver 12.0 mAh cm^−2^ initial areal capacity, maintaining 8.2 mAh cm^−2^ after 50 cycles. This approach provides valuable insights for designing high‐performance transition metal dichalcogenide catalysts in conversion‐type batteries.

## Conflict of Interest

The authors declare no conflict of interest.

## Supporting information



Supporting Information

## Data Availability

The data that support the findings of this study are available from the corresponding author upon reasonable request.

## References

[1] H. Dong , X. Gao , J. Zhu , B. Xiong , H. He , M. Ouyang , G. He , H. Li , Z. Lin , Mater. Today Energy 2025, 50, 101864.

[2] W. Yan , J. L. Yang , X. Xiong , L. Fu , Y. Chen , Z. Wang , Y. Zhu , J. W. Zhao , T. Wang , Y. Wu , Adv. Sci. 2022, 9, 2202204. 10.1002/advs.202202204 PMC9443453 35748192

[3] W. Liu , Y. Zhao , C. Yi , W. Hu , J. Xia , Y. Li , J. Liu , Energy Environ. Mater. 2024, 7, 12719.

[4] Y. Wu , J. Huang , Z. Zhang , D. Chen , H. Yu , F. Ma , X. Zhang , Y. Chen , Energy Mater. 2025, 5, 500027.

[5] K. Du , E. H. Ang , X. Wu , Y. Liu , Energy Environ. Mater. 2022, 5, 1012.

[6] F. Liang , Q. Deng , S. Ning , H. He , N. Wang , Y. Zhu , J. Zhu , Adv. Sci. 2024, 11, 2403391. 10.1002/advs.202403391 PMC11348264 38925593

[7] X. Cui , X. Wang , Q. Pan , Energy Mater. 2023, 3, 300034.

[8] J.‐L. Yang , D.‐Q. Cai , X.‐G. Hao , L. Huang , Q. Lin , X.‐T. Zeng , S.‐X. Zhao , W. Lv , ACS Nano 2021, 15, 11491. 34190550 10.1021/acsnano.1c01250

[9] W. Yan , J. Xian , S. Huang , Y. Leng , Q. Liu , T. Xiao , Y. Zhao , P. Yang , Y. Wu , Energy Storage Mater. 2025, 76, 104150.

[10] B. Lin , Y. Zhang , W. Li , J. Huang , Y. Yang , S. W. Or , Z. Xing , S. Guo , eScience 2024, 4, 100180.

[11] J. Chou , Y.‐H. Wang , W.‐P. Wang , S. Xin , Y.‐G. Guo , J. Electrochem. 2023, 29, 2217009.

[12] M. Chen , Q. Fan , K. Chen , E. Majkova , Q. Huang , K. Liang , Carbon Neutralization 2024, 3, 493.

[13] V. P. Nguyen , J.‐H. Kim , S.‐M. Lee , Mater. Today Energy 2023, 38, 101451.

[14] M. Yan , X. Cheng , L. Shi , Y. Pan , P. He , Z. Zhang , Z. Lun , Y. Fu , H. Zhang , Chem. Eng. J. 2023, 455, 140616.

[15] S. Xie , X. Chen , L. Wang , G. Zhang , H. Lv , G. Cai , Y.‐R. Lu , T.‐S. Chan , J. Zhang , J. Dong , H. Jin , X. Kong , J. Lu , S. Jin , X. Wu , H. Ji , eScience 2024, 4, 100222.

[16] L. Wang , H. Shi , Y. Xie , Z. S. Wu , Carbon Neutralization 2023, 2, 262.

[17] G. Babu , N. Masurkar , H. Al Salem , L. M. R. Arava , J. Am. Chem. Soc. 2016, 139, 171. 28001059 10.1021/jacs.6b08681

[18] J. Xie , J. Zhang , S. Li , F. Grote , X. Zhang , H. Zhang , R. Wang , Y. Lei , B. Pan , Y. Xie , J. Am. Chem. Soc. 2013, 135, 17881. 24191645 10.1021/ja408329q

[19] H. Li , M. Chuai , X. Xiao , Y. Jia , B. Chen , C. Li , Z. Piao , Z. Lao , M. Zhang , R. Gao , B. Zhang , Z. Han , J. Yang , G. Zhou , J. Am. Chem. Soc. 2023, 145, 22516. 37788438 10.1021/jacs.3c07213

[20] C. Dong , C. Ma , C. Zhou , Y. Yu , J. Wang , K. Yu , C. Shen , J. Gu , K. Yan , A. Zheng , M. Gong , X. Xu , L. Mai , Adv. Mater. 2024, 36, 407070. 10.1002/adma.202407070 39091051

[21] M. Yan , X. Cheng , L. Gong , Z. Lun , P. He , L. Shi , C. Liu , Y. Pan , Chem. Eng. J 2023, 475, 146417.

[22] G. Liu , T. Yan , Y. Zhang , P. Zeng , B. Wang , C. Yuan , C. Cheng , L. Wang , X. Liu , J. Zeng , L. Zhang , Nano Lett. 2024, 24, 15973. 39651781 10.1021/acs.nanolett.4c04139

[23] W. Wang , W. Zhang , R. Yu , F. Qiao , J. Wang , J. Wang , Q. An , ACS Nano 2024, 18, 35286. 10.1021/acsnano.4c11147 39680707

[24] H. Li , R. Yu , H. Chen , J. Hu , J. Zhang , G. Hou , Q. Chen , J. Lu , Y. Tang , ACS Energy Lett. 2024, 10, 168.

[25] S. Li , Y. Liu , X. Zhao , Q. Shen , W. Zhao , Q. Tan , N. Zhang , P. Li , L. Jiao , X. Qu , Adv. Mater. 2021, 33, 2007480. 10.1002/adma.202007480 33598960

[26] J.‐L. Yang , D.‐Q. Cai , Q. Lin , X.‐Y. Wang , Z.‐Q. Fang , L. Huang , Z.‐J. Wang , X.‐G. Hao , S.‐X. Zhao , J. Li , Nano Energy 2022, 91, 106669.

[27] W. Ding , L. Hu , J. Dai , X. Tang , R. Wei , Z. Sheng , C. Liang , D. Shao , W. Song , Q. Liu , M. Chen , X. Zhu , S. Chou , X. Zhu , Q. Chen , Y. Sun , S. X. Dou , ACS Nano 2019, 13, 1694. 30649862 10.1021/acsnano.8b07744

[28] X. Wang , Y. Zhang , H. Si , Q. Zhang , J. Wu , L. Gao , X. Wei , Y. Sun , Q. Liao , Z. Zhang , K. Ammarah , L. Gu , Z. Kang , Y. Zhang , J. Am. Chem. Soc. 2020, 142, 4298. 31999446 10.1021/jacs.9b12113

[29] J.‐L. Yang , P. Yang , D.‐Q. Cai , Z. Wang , H. J. Fan , Nano Lett. 2023, 23, 4000. 37125765 10.1021/acs.nanolett.3c00787

[30] R. Liu , J. Feng , R. Tang , T. Meng , Chem. Eng. J. 2023, 468, 143766.

[31] X. Jin , T. Lee , A. Soon , S. J. Hwang , Adv. Funct. Mater. 2024, 34, 2316446.

[32] K. Liao , L. Chen , R. Meng , Y. Feng , S. Meng , H. Lu , J. Ma , C. Peng , C. Zhang , J. Yang , J. Am. Chem. Soc. 2024, 146, 12020. 38651300 10.1021/jacs.4c01550

[33] X. Fan , R. R. Gaddam , N. A. Kumar , X. S. Zhao , Adv. Energy Mater. 2017, 7, 1700317.

[34] J.‐L. Yang , S.‐X. Zhao , Y.‐M. Lu , X.‐T. Zeng , W. Lv , G.‐Z. Cao , J. Mater. Chem. A 2020, 8, 231.

[35] B. Hu , L. Mai , W. Chen , F. Yang , ACS Nano 2009, 3, 478. 19236088 10.1021/nn800844h

[36] B. Zhao , Z. Cong , Z. Cheng , Z. Sun , B. Yuan , F. Shen , X. Han , ACS Appl. Energy Mater. 2021, 4, 10252.

[37] W. Yan , X. Gao , J. L. Yang , X. Xiong , S. Xia , W. Huang , Y. Chen , L. Fu , Y. Zhu , Y. Wu , Small 2022, 18, 2106679. 10.1002/smll.202106679 35060309

[38] F. Y. Fan , W. C. Carter , Y. M. Chiang , Adv. Mater. 2015, 27, 5203. 26257297 10.1002/adma.201501559

[39] W. Hua , T. Shang , H. Li , Y. Sun , Y. Guo , J. Xia , C. Geng , Z. Hu , L. Peng , Z. Han , C. Zhang , W. Lv , Y. Wan , Nat. Catal. 2023, 6, 174.

[40] J.‐Q. Huang , T.‐Z. Zhuang , Q. Zhang , H.‐J. Peng , C.‐M. Chen , F. Wei , ACS Nano 2015, 9, 3002. 25682962 10.1021/nn507178a

