# Supplementary material for: Oxygen‐Doped MoS2 with Expanded Interlayer Spacing for Rapid and Stable Polysulfide Conversion
Source: Adv Sci (Weinh). 2025 Apr 7;12(26):2502834. doi: 10.1002/advs.202502834 (PMC12245086; doi:10.1002/advs.202502834)
Supplement: Supplementary file 1 — Supporting Information [file ADVS-12-2502834-s001.pdf]

ADVANCED SCIENCE
Open Access

Supporting Information

for *Adv. Sci.*, DOI 10.1002/advs.202502834

Oxygen-Doped MoS_2 with Expanded Interlayer Spacing for Rapid and Stable Polysulfide Conversion

Wenqi Yan, Jinglin Xian, Shunan Zhang, Jiarui Zhang, Kaisi Liu, Jin-Lin Yang, Feng Tao, Ruiping Liu, Qi Liu* and Peihua Yang**

Supporting Information

1. Methods

2. Supplementary Figures

3. Supplementary Tables

4. Supplementary References

Methods

***Preparation of E-MoS_2 and MoS_2 nanosheets*:** A homogeneous precursor solution was prepared by dissolving 1.359 g ammonium molybdate tetrahydrate ($(NH_4)_6Mo_7O_{24}\cdot 4H_2O$, Aladdin, 99.0 %) and 2.512 g thiourea (NH_2CSNH_2, Aladdin, 99.0 %) in 38 mL deionized water under sonication. The solution was hydrothermally treated at 180 °C for 24 h, yielding oxygen-doped MoS_2 (E-MoS_2) with structural defects. Pristine MoS_2 was synthesized analogously at 220 °C. Both products were washed with deionized water and freeze-dried.

***Preparation of Graphene/Sulfur (G/S) composite*:** Graphene powder (Sixth Element Inc., Changzhou, China) was annealed at 900 °C under Ar for 2 h. The activated graphene was mixed with sulfur powder (1:3 mass ratio), ground for 40 min, and heat-treated at 155 °C for 24 h.

***Materials characterization*:** Crystal structures were analyzed via X-ray diffraction (XRD, Bruker D8 Advance, Cu Kα, λ = 0.15418 nm, 10° min^{-1}). Morphology and elemental distribution were assessed using SEM (ZEISS SUORA55) with an energy dispersive spectrometer (EDS). Atomic-scale imaging was performed via HAADF-STEM and HRTEM (FEI TECNAI G2 F30, 300 kV) coupled with EDS. Surface area and pore size distribution were determined by N_2 adsorption-desorption (Quantachrome Autosorb iQ3). X-ray absorption fine structure (XAFS) spectra were acquired at SSRF (13SSW) and BSRF (1W1B) beamlines, processed using Athena software. Raman spectra (Horiba HR800), XPS (Thermo Fisher ESCALAB 250Xi), and UV-vis absorption (Shimadzu UV-2550) were conducted for electronic and optical analyses.

***Li_2S_6 visualized adsorption tests*:** The Li_2S_6 solution was prepared by mixing Li_2S and sulfur powder in a 1:5 molar ratio, then dissolving the mixture in a DOL/DME solution and stirring at 50 °C overnight. Subsequently, 30 mg of the sample (graphene, MoS_2, or E-MoS_2) was dispersed into the prepared 2 mM Li_2S_6 solution (3 mL) and allowed to rest for 1 hour.

***Li_2S_6 symmetric cells*:** In this section, a 0.2 M Li_2S_6 solution was prepared in the

electrolyte (1 M LiTFSI in DOL/DME, 1:1 by volume). The electrodes were fabricated by loading the samples (E-MoS_2 and MoS_2) onto carbon paper (CP). A symmetric cell was assembled using the prepared electrodes, with both the working and counter electrodes containing the same active material, and 40 μL of the Li_2S_6 solution. Cyclic voltammetry (CV) tests were performed within a voltage window of −1 to 1 V (vs. Li/Li^+).

***Li_2S nucleation/dissolution tests*:** Li_2S_8 catholyte (0.2 M) was prepared by stirring Li_2S and sulfur (1:7 molar ratio) in DOL/DME at 50 °C for 24 h. For nucleation tests, catalyst-coated CP electrodes (~3 mg cm^{-2}) were discharged to 2.09 V (0.134 mA) and held at 2.08 V until current decayed to <0.01 mA.[1]

***Li_2S dissolution test*:** The cells assembled as described were initially discharged to 1.80 V at a constant current of 0.134 mA, followed by a further discharge at the same current until the complete transformation of LiPSs into solid Li_2S. The cells were then charged potentiostatically at 2.40 V to facilitate the oxidation of solid Li_2S back into soluble LiPSs.[2]

***Cell assembly and electrochemical measurements*:** Cathodes were prepared by blending graphene/sulfur composite (80 wt%), E-MoS_2 or MoS_2 catalyst (5 wt%), Super P conductive carbon (5 wt%), and PVDF binder (10 wt%) in N-methyl-2-pyrrolidone (NMP) to form a homogeneous slurry, which was stirred for 6–8 h. For high-sulfur-loading cathodes, carboxymethyl cellulose (CMC) replaced PVDF in aqueous slurry formulations. The slurry was uniformly coated onto carbon-coated aluminum foil using a doctor blade technique, followed by vacuum drying at 60 °C for 12 h. Circular electrodes (12 mm diameter) were punched from the dried foil, achieving sulfur loadings of 1.1-1.2 mg cm^{-2}. The graphene/sulfur cathode, graphene/ MoS_2/sulfur cathode and graphene/ E-MoS_2/sulfur are abbreviated as G/S, G/ MoS_2/S, and G/E-MoS_2/S, respectively.

CR2025 coin batteries were assembled in an Ar-filled glovebox using lithium metal anodes, Celgard 2400 separators, and the prepared cathodes. The electrolyte consisted of 1 M lithium bis(trifluoromethanesulfonyl)imide (LiTFSI) in a 1:1 v/v

mixture of 1,3-dioxolane (DOL) and 1,2-dimethoxyethane (DME) with 2 wt% $LiNO_3$ additives. Galvanostatic charge/discharge cycling was performed on a LAND CT2001A system (Wuhan Land Electronics) between 1.7–2.8 V vs. Li/Li^+, with specific capacities normalized to sulfur mass (1 C = 1675 mA g^{-1}). Cyclic voltammetry (CV) and electrochemical impedance spectroscopy (EIS) were conducted using a Gamry Interface 1000 workstation (Germany) at ambient temperature.

***The shuttle constant k_s*:** The shuttle constants (k_s, h^{-1}) of different samples were determined using the charge-discharge curves at 0.2 C using the following formulae:[3]

$$\frac{k_S q_H[\mathrm{s}_{\mathrm{total}}]}{I_C} = f_C \tag{1}$$

$$H_{OC} = \frac{Q_H^{\mathrm{applied}} - Q_H^{acc}}{Q_H^{\mathrm{acc}}} = -\frac{1}{f_C}\ln(1 - f_C) - 1 \tag{2}$$

where f_C is the charge-shuttle factor; [S_{total}]is the total sulfur mass in the cell, which is is ~1.2 mg for all samples in this work; q_H is the high plateau sulfur specific capacity (419 mAh g according to a previous report);[3] I_C is the value of current at the current density of 0.2 C; $Q_H^{applied}$ is the applied high plateau charge capacity and is simplified here to the maximum charge capacity; Q_H^{acc} means the high plateau accumulated charge capacity and is simplified here to the practical charge capacity.

***Computational methods*:** All DFT calculations were performed using the Vienna ab initio simulation package (VASP) with projector augmented wave potentials.[4] The generalized gradient approximation proposed by Perdew-Burke-Ernzerhof (PBE) was selected for the exchange-correlation potential. DFT-D3 method was chosen to describe the Van der Waals correction. The computational setup was optimized for precision, with a kinetic energy cutoff of 500 eV, which ensures a sufficiently converged representation of the plane-wave basis set. The force convergence criterion was set to 0.05 eV/Å, ensuring that the atomic positions were relaxed to a high degree of accuracy during geometry optimizations. A 1×2×1 Gamma-centered k-point mesh was employed

for sampling the first Brillouin zone, striking a balance between computational efficiency and the accurate representation of the electronic structure for the system under study. Meanwhile, a vacuum region of approximately 15 Å was implemented to prevent interactions between adjacent periodic images. The visualization of molecular models and the processing of differential charge densities were performed by using VESTA. The migration energy barriers of lithium ions between the layers of 2H-MoS_2 and 2H-$MoS_{0.75}O_{0.15}$ were calculated using the Climbing Image Nudged Elastic Band (Cl-NEB) method to determine the energy barriers for ion transport across the interface.

The binding energy (E_b) was calculated by the following equation:

$$E_b = E_{(*\mathrm{Li_2S}_x)} - E_{(*)} - E_{(\mathrm{Li_2S}_x)} \quad (3)$$

where $E_{(*\mathrm{Li_2S}_x)}$ is the total energy obtained from self-consistency of the optimized whole structure, $E_{(*)}$ is the self-consistent energy obtained by deleting polysulfide after optimizing the overall structure (i.e., 2H-MoS_2, 2H-$MoS_{0.75}O_{0.15}$), and $E_{(\mathrm{Li_2S}_x)}$ (x=2, 6) is the self-consistent energy obtained by deleting the bare surface after optimizing the overall structure.

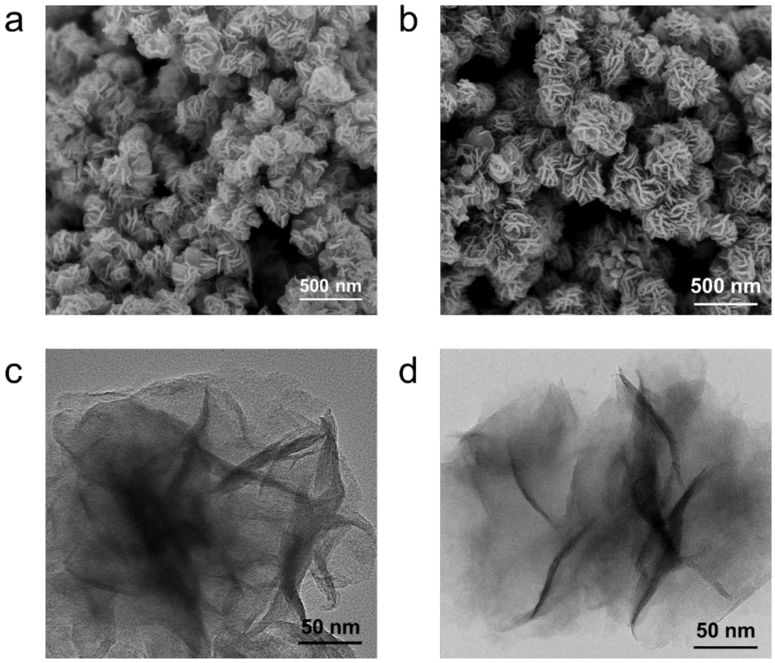

Figure S1. (a, b) SEM and (c, d) TEM of (a, c) MoS_2 and (c, d) E-MoS_2.

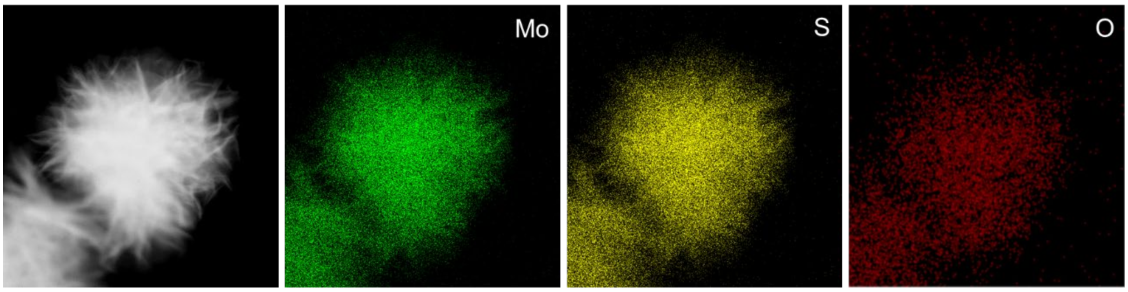

Figure S2. STEM image, and corresponding elemental mapping images of E-MoS_2.

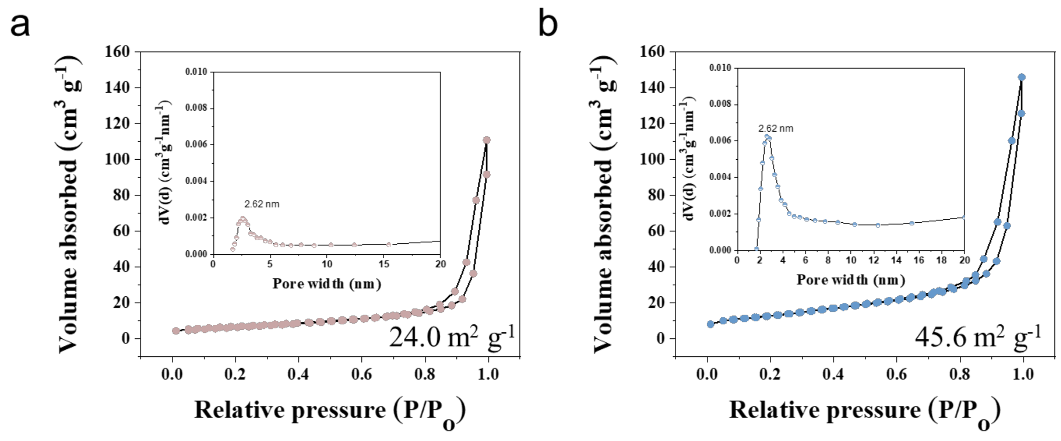

Figure S3. Nitrogen adsorption isotherms (at 77 K) of the (a) MoS_2 and (b) E-MoS_2.

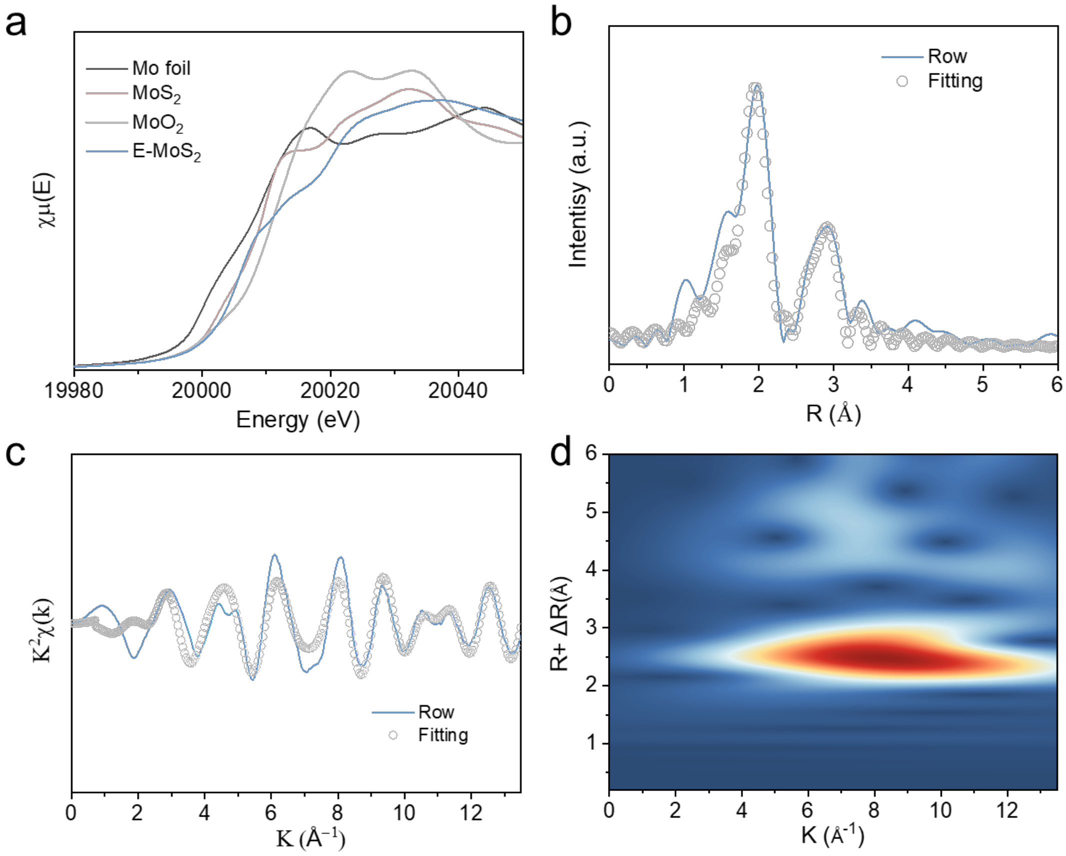

Figure S4. (a) XANES spectra at the Mo K-edge for the E-MoS_2, MoO_2, MoS_2 and Mo foil. FT-EXAFS fitting curves of the E-MoS_2 at Mo K-edge in (b) R-space and (c) K-space. (d) WT-EXAFS spectra of the Mo foil.

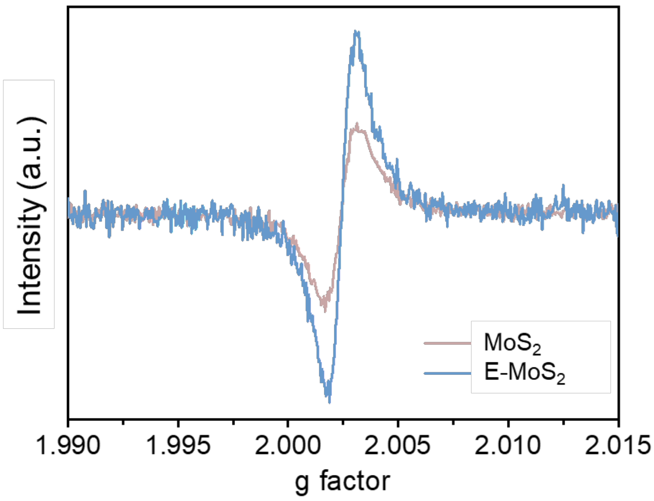

Figure S5. EPR spectra of MoS_2 and E-MoS_2.

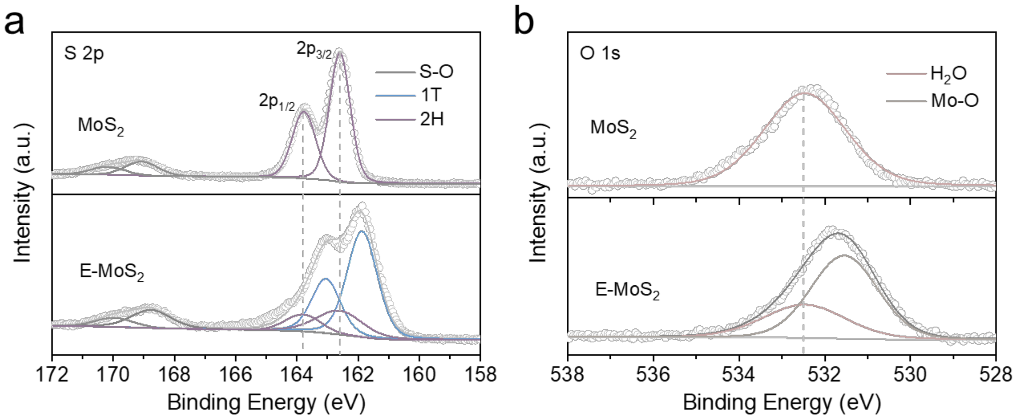

Figure S6. (a) S 2p and (b) O 1s XPS spectra of MoS_2 and E-MoS_2.

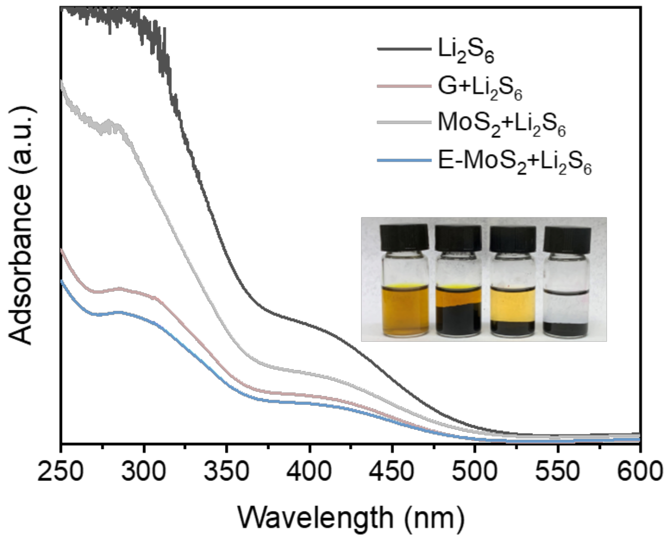

Figure S7. UV-vis absorption spectra of Li_2S_6 before and after adsorption by various materials for 12 h (inset figure: optical images of the above solutions).

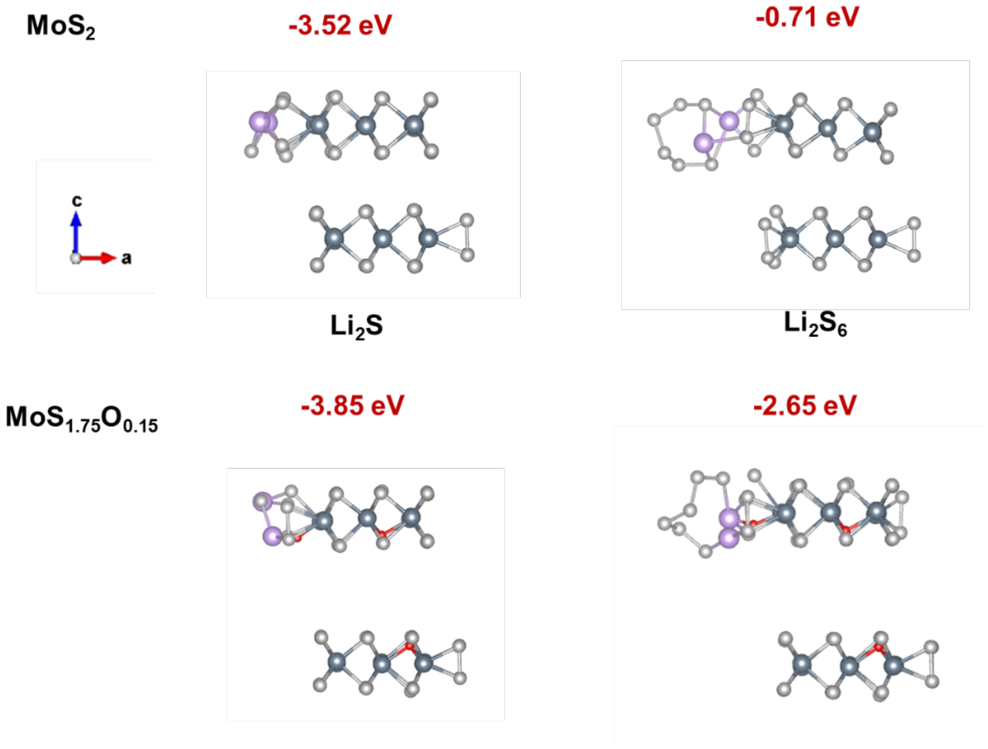

Figure S8. Interaction energy between sulfur species (Li_2S and Li_2S_6) and various samples.

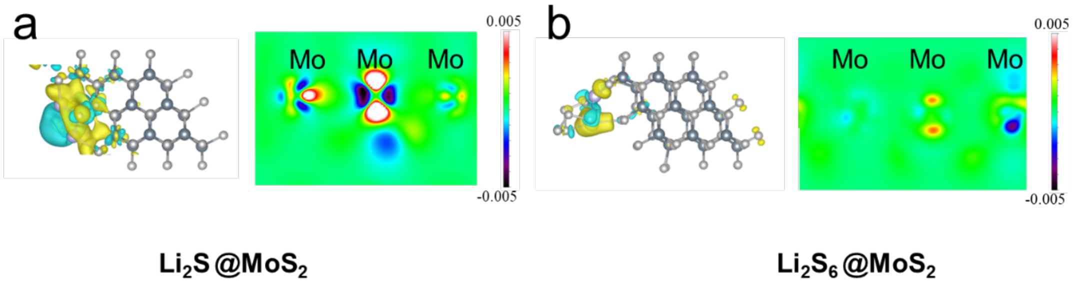

Figure S9. Electron-density difference analysis of (a) Li_2S and (b) Li_2S_6 on the surface of MoS_2.

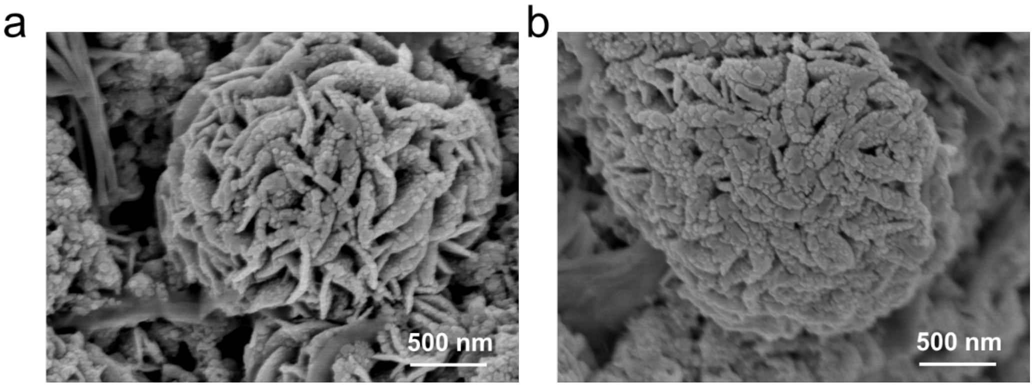

Figure S10. SEM images of (a) MoS_2 and (b) E- MoS_2 electrodes after Li_2S deposition.

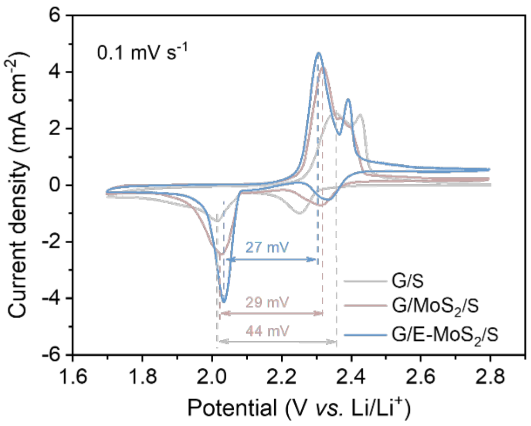

Figure S11: CV curves of different Li-S batteries at the scan rate of 0.1 mV s^{-1}.

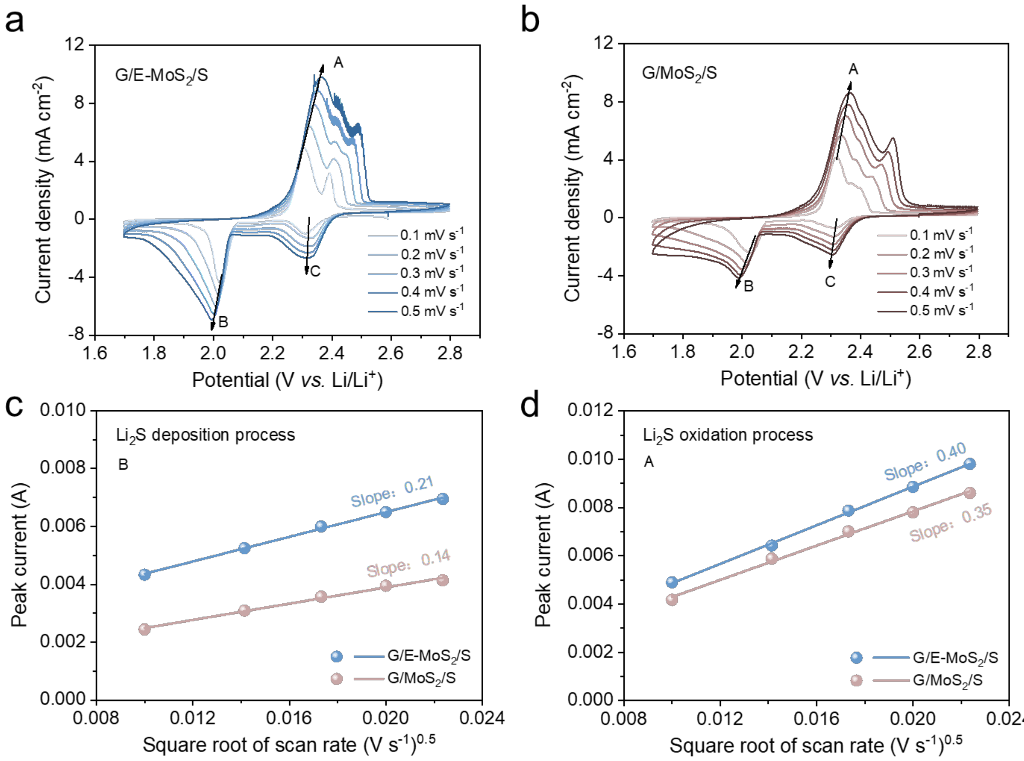

Figure S12. CV curves at various scan rates and the corresponding linear fits of the peaks for Li_2S deposition and oxidation process.

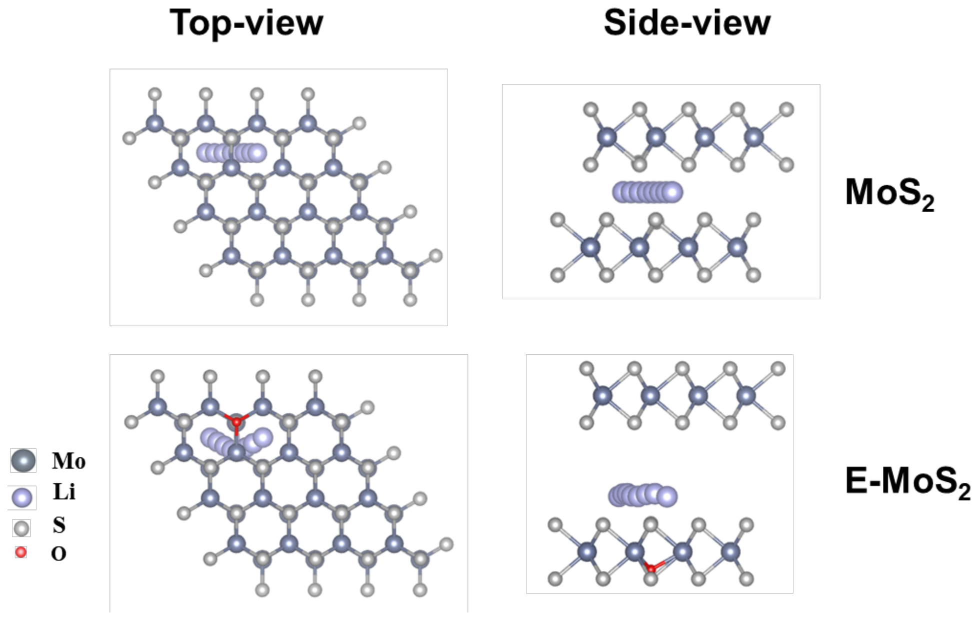

Figure S13. Li^+ diffusion path on MoS_2 (a) and E-MoS_2.

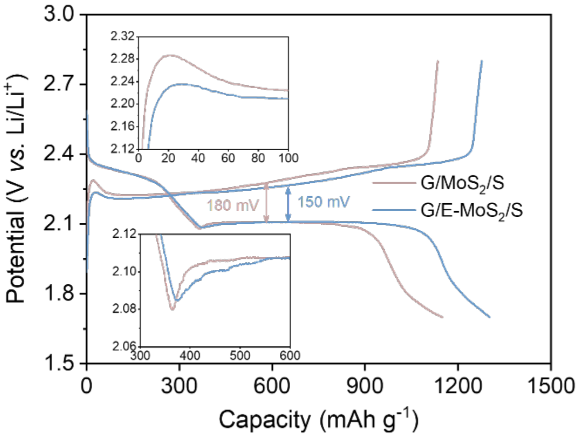

Figure S14. The comparison of the discharge-charge curves of different cells at 0.2 C. Insets highlight the reduced activation and nucleation energy barriers for Li_2S in E-MoS_2 electrode.

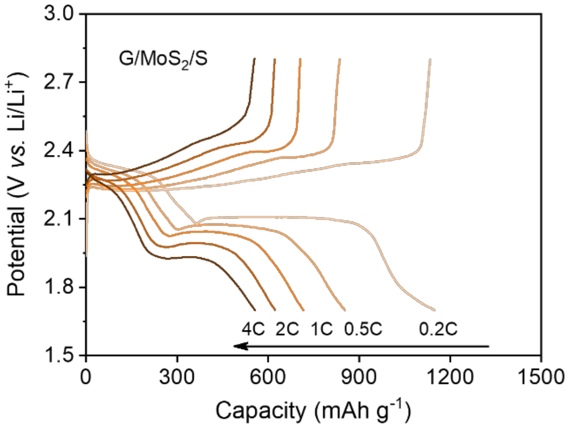

Figure S15. The corresponding galvanostatic discharge-charge curves of rate performance.

Table S1. Strategies for expanded MoS_2 in different applications.

**Type of MoS_2**	**Strategy**	**Preparation method**	**Interlayer spacing**		**Pillar**	**Application**	**Reference**
			**Original**	**Expanded**			
E-MoS_2	O-doping	One-step hydrothermal	0.63	0.95	No	Li–S batteries	This work
N-MoS_2	N-Doping	Hydrothermal and annealing	0.62	0.65	No	Li–S batteries	[5]
MoS_2-HNS	Self-templating	Hydrothermal and annealing	0.62	0.66	No	Li-ion batteries	[6]
E-MG	Glucose-mediated	Hydrothermal and annealing	0.62	1.01	No	Mg^{2+}/Li^{+} batteries	[7]
ES-1T-MoS_2	Insert CTAB molecules	One-step hydrothermal method	0.63	1.00	Yes	Aqueous Ca-ion batteries	[8]
MoS_2/g-CM	N-doped C intercalation	Hydrothermal and annealing	0.64	0.98	Yes	Li-ion batteries	[9]
Etched MoS_2	S-vacancies etching	Hydrothermal and H_2O_2 etching	0.64	0.68	No	Hydrogen evolution reaction	[10]
MoS_2-C	SCN^- intercalation	Hydrothermal precursor modulation	0.64	0.99	Yes	Aqueous NH_4^+ batteries	[11]
Fe-MoS_2-C	S-Fe-C intercalation	Hydrothermal and annealing	0.64	0.93	Yes	Li–S batteries	[12]
LE- MoS_2	Carbon intercalation	Hydrothermal and annealing	0.98	1.04	Yes	Li–S batteries	[13]

Table S2. Battery components and setup information.

**Components**	**Material and its composition**
Active material	Sulfur (S); 80% Graphene/Sulfur (G/S) composite cathode (G:S=3:1, Theoretical capacity of S: 1675 mAh g^{-1})
Additive	5% E-MoS_2 or MoS_2 catalyst and 5 wt% Super P conductive carbon
Binder	10% Polyvinylidene fluoride (PVDF)
Electrolyte	1 M LiTFSI in DOL/DME (1:1 v/v) with 2 wt% $LiNO_3$ additive
Separator	Celgard 2400 separators
Current collector	Aluminum foil
Full cell	Anode: Lithium foil<br>Cathode: Graphene/Sulfur (G/S) composite

Table S3. Summary of cycling and rate performance.

**Condition**	**Performance**
Initial capacity (mAh/g)	1086 mAh g^{-1} (0.5 C); 590 mAh g^{-1} (2 C)
Capacity achieved (mAh/g) @rate	1302, 1029, 913, 812, and 665 mAh g^{-1} at 0.2, 0.5, 1, 2 and 4 C, respectively.
Number of cycles tested	200 (0.2C); 600 (2 C)
Capacity retention	773 mAh g^{-1} (0.5 C); 638 mAh g^{-1} (2 C)
High loading tests	Initial: 5.7 mAh cm^{-2}; 4.0 mAh cm^{-2} after 50 cycles (6 mg_s cm^{-2} at 0.2 C; E/S= 6 μL mg^{-1}) Initial: 12.0 mAh cm^{-2}; 8.2 mAh cm^{-2} after 50 cycles. (12mg_s cm^{-2} at 0.2 C; E/S= 4 μL mg^{-1})

References

[1] B. Q. Li, L. Kong, C. X. Zhao, Q. Jin, X. Chen, H. J. Peng, J. L. Qin, J. X. Chen, H. Yuan, Q. Zhang, J. Q. Huang, *InfoMat* **2019**, 1, 533.
[2] J.-L. Yang, D.-Q. Cai, X.-G. Hao, L. Huang, Q. Lin, X.-T. Zeng, S.-X. Zhao, W. Lv, *ACS Nano* **2021**, 15, 11491.
[3] Y. V. Mikhaylik, J. R. Akridge, *J. Electrochem. Soc.* **2004**, 151, A1969.
[4] D. J. G Kresse, *Phys. Rev. B* **1999**, 59, 1758.
[5] M. Chen, N. Wang, W. Zhou, X. Zhu, Q. Wu, M. H. Lee, D. Zhao, S. Ning, M. An, L. Li, *Small* **2023**, 19, 2303015.
[6] Y. Wang, L. Yu, X. W. Lou, *Angew. Chem. Int. Ed.* **2016**, 55, 7423.
[7] X. Fan, R. R. Gaddam, N. A. Kumar, X. S. Zhao, *Adv. Energy Mater.* **2017**, 7, 1700317.
[8] W. Wang, W. Zhang, R. Yu, F. Qiao, J. Wang, J. Wang, Q. An, *ACS Nano* **2024**, 18, 35286.
[9] K. Liao, L. Chen, R. Meng, Y. Feng, S. Meng, H. Lu, J. Ma, C. Peng, C. Zhang, J. Yang, *J. Am. Chem. Soc.* **2024**, 146, 12020.
[10] X. Wang, Y. Zhang, H. Si, Q. Zhang, J. Wu, L. Gao, X. Wei, Y. Sun, Q. Liao, Z. Zhang, K. Ammarah, L. Gu, Z. Kang, Y. Zhang, *J. Am. Chem. Soc.* **2020**, 142, 4298.
[11] H. Li, R. Yu, H. Chen, J. Hu, J. Zhang, G. Hou, Q. Chen, J. Lu, Y. Tang, *ACS Energy Lett.* **2024**, 10, 168.
[12] G. Liu, T. Yan, Y. Zhang, P. Zeng, B. Wang, C. Yuan, C. Cheng, L. Wang, X. Liu, J. Zeng, L. Zhang, *Nano Lett.* **2024**, 24, 15973.
[13] Y. Pan, L. Gong, X. Cheng, Y. Zhou, Y. Fu, J. Feng, H. Ahmed, H. Zhang, *ACS Nano* **2020**, 14, 5917.
